# The role of antigen presenting cells in the induction of HIV-1 latency in resting CD4^+^ T-cells

**DOI:** 10.1186/s12977-015-0204-2

**Published:** 2015-09-11

**Authors:** Nitasha A. Kumar, Karey Cheong, David R. Powell, Candida da Fonseca Pereira, Jenny Anderson, Vanessa A. Evans, Sharon R. Lewin, Paul U. Cameron

**Affiliations:** Department of Infectious Diseases, Alfred Hospital and Monash University, Melbourne, VIC 3004 Australia; Centre for Biomedical Research, Burnet Institute, Melbourne, VIC 3004 Australia; Victorian Life Science Computational Initiative, Parkville, 3010 Australia; Monash Bioinformatics Platform, Monash University, Clayton, 3800 Australia; Doherty Institute for Infection and Immunity, University of Melbourne, Melbourne, 3010 Australia

**Keywords:** Dendritic cells, Monocytes, B-cells, HIV Latency, Resting CD4^+^ T-cells, Antigen presenting cells, APC, Viral reservoir, Latency induction, Post-integration latency

## Abstract

**Background:**

Combination antiretroviral therapy (cART) is able to control HIV-1 viral replication, however long-lived latent infection in resting memory CD4^+^ T-cells persist. The mechanisms for establishment and maintenance of latent infection in resting memory CD4^+^ T-cells remain unclear. Previously we have shown that HIV-1 infection of resting CD4^+^ T-cells co-cultured with CD11c^+^ myeloid dendritic cells (mDC) produced a population of non-proliferating T-cells with latent infection. Here we asked whether different antigen presenting cells (APC), including subpopulations of DC and monocytes, were able to induce post-integration latent infection in resting CD4^+^ T-cells, and examined potential cell interactions that may be involved using RNA-seq.

**Results:**

mDC (CD1c^+^), SLAN^+^ DC and CD14^+^ monocytes were most efficient in stimulating proliferation of CD4^+^ T-cells during syngeneic culture and in generating post-integration latent infection in non-proliferating CD4^+^ T-cells following HIV-1 infection of APC-T cell co-cultures. In comparison, plasmacytoid DC (pDC) and B-cells did not induce latent infection in APC-T-cell co-cultures. We compared the RNA expression profiles of APC subpopulations that could and could not induce latency in non-proliferating CD4^+^ T-cells. Gene expression analysis, comparing the CD1c^+^ mDC, SLAN^+^ DC and CD14^+^ monocyte subpopulations to pDC identified 53 upregulated genes that encode proteins expressed on the plasma membrane that could signal to CD4^+^ T-cells via cell–cell interactions (32 genes), immune checkpoints (IC) (5 genes), T-cell activation (9 genes), regulation of apoptosis (5 genes), antigen presentation (1 gene) and through unknown ligands (1 gene).

**Conclusions:**

APC subpopulations from the myeloid lineage, specifically mDC subpopulations and CD14^+^ monocytes, were able to efficiently induce post-integration HIV-1 latency in non-proliferating CD4^+^ T-cells in vitro. Inhibition of key pathways involved in mDC-T-cell interactions and HIV-1 latency may provide novel targets to eliminate HIV-1 latency.

**Electronic supplementary material:**

The online version of this article (doi:10.1186/s12977-015-0204-2) contains supplementary material, which is available to authorized users.

## Background

Despite the successes of cART in the reduction of morbidity and mortality world wide, treatment is required life long. HIV-1 persists in individuals on cART in resting CD4^+^ T-cells as latent infection [1–3]. Latency occurs when viral DNA is integrated within the host genome and remains transcriptionally silent. Latent infection of resting CD4^+^ T-cells therefore represents the major barrier to HIV-1.

It remains unclear how latency is established in resting CD4^+^ T-cells in vivo. Initial studies in vitro, showed that direct HIV-1 infection of resting CD4^+^ T-cells isolated from peripheral blood was inefficient and integration rarely occurred due to incomplete reverse transcription, reduced nuclear import of the viral DNA and/or limited integration within the host genome [4–6]. However, in vitro latent infection can occur following the reversion of a HIV-1 infected, activated CD4^+^ T-cell to a resting state [7–10]. Alternatively, latent infection can also occur following the direct infection of a resting CD4^+^ T-cell exposed to high viral titers and spinoculation [11, 12], chemokines [13] or co-culture with other cell types [14, 15].

As professional APCs, DC interact with HIV-1 during initial infection at vaginal and rectal mucosa sites and in blood. Langerhan cells (LC) and dermal (D)DC at mucosa and, bone marrow derived classical or myeloid (m)DC and plasmacytoid (p)DC in blood are able to interact with T-cells, but their role in the establishment and maintenance of HIV-1 latency remain unclear [16–18]. Blood derived mDC subpopulations differ from tissue DC. CD141^+^ and CD1c^+^ mDC are both found as resident cells in tissue (lymph node (LN), spleen, lungs), skin and, as more mature cells, circulating through the lymphatics to the LN [19–22]. SLAN^+^ DC represent a subpopulation of monocytic cells with increased potential to secrete pro-inflammatory cytokines and develop a DC phenotype, however precise residence remains unknown [23, 24]. CD14^+^ monocytes represent DC and macrophage precursors in blood [Reviewed in 25], and were also tested for their ability to establish latent infection in resting CD4^+^ T-cells.

We have previously developed an in vitro co-culture model demonstrating that CD11c^+^ myeloid dendritic cells (mDC) induce post-integration latency in non-proliferating memory CD4^+^ T-cells. Here we demonstrate that in addition to the mDC subsets (CD1c^+^, SLAN^+^ and CD141^+^), CD14^+^ monocytes were also able to induce post-integration HIV-1 latency in non-proliferating CD4^+^ T-cells. In comparison, T-cells co-cultured with pDC and B-cells were inefficient in the induction of latency. Using RNA-seq and Illumina gene expression microarrays, we also identified potential mediators of latent infection expressed by APC that could induce latency in the non-proliferating CD4^+^ T-cells during APC-T cell interactions.

## Results

### Monocytes are able to induce latency in resting CD4^+^ T-cells

**Fig. 1:**
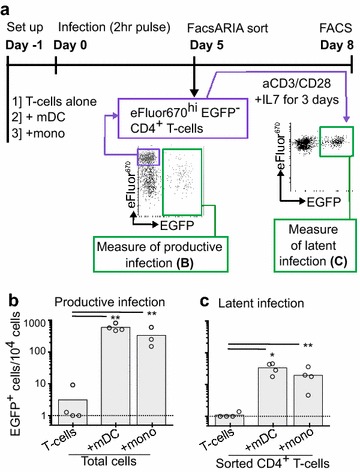
Monocyte induced latency in non-proliferating CD4^+^ T-cells. **a** Resting CD4^+^ T-cells were labeled with eFluor670 and cultured alone, with bulk myeloid (m)DC or bulk monocytes (mono) at a ratio of 10:1. Following 24 h of culture, APC-T-cell co-cultures were infected with NL(AD8)Δnef-EGFP. **b** At day 5 post-infection productive infection was determined by quantification of EGFP in total cells and, non-proliferating, non-productively infected (eFluor670^hi^EGFP^−^) cells were sorted using flow cytometry and reactivated to determine frequency of latent infection. **c** Sorted non-proliferating (eFluor670^hi^EGFP^−^) CD4^+^ T-cells were stimulated with phytohaemagglutinin (PHA) stimulated feeder PBMC for 5 days and EGFP quantified as a measure of HIV-1 latency. *Columns* represent the median of the log transformed values, *open circles* represent individual donors. *p ≤ 0.05, **p ≤ 0.005 as determined by paired students t-test

We have previously reported that mDC, but not pDC, are able to efficiently induce post-integration latent infection in resting CD4^+^ T-cells using an in vitro DC-T-cell co-culture model [14]. However, mDC and their subpopulations represent only a small proportion of peripheral blood mononuclear cells (PBMC) compared to monocytes, which represent a precursor to some DC and macrophage subpopulations. Therefore we compared monocytes and mDC isolated from healthy donors for their ability to induce latent infection in resting CD4^+^ T-cells (Fig. 1). eFluor670 labeled resting CD4^+^ T-cells were cultured alone, with CD11c^+^ mDC or bulk monocytes and infected with an R5 using virus that expresses enhanced green fluorescent protein (EGFP). Similar to mDC, monocytes were able to induce productive infection in CD4^+^ T-cells, as measured by total EGFP expression at day 5 post-infection (Fig. 1b). At day 5 post-infection non-proliferating (eFluor670^hi^EGFP^−^) CD4^+^ T-cells were sorted and cultured with phytohemagglutinin (PHA)-stimulated feeder peripheral blood mononuclear cells (PBMC), where the number of EGFP^+^ cells was quantified by flow cytometry as a surrogate marker of inducible latent infection. CD14^+^ monocytes were also able to significantly increase the induction of latent infection in non-proliferating cells (p > 0.05; Fig. 1c).

### Isolation of functional APC

**Fig. 2:**
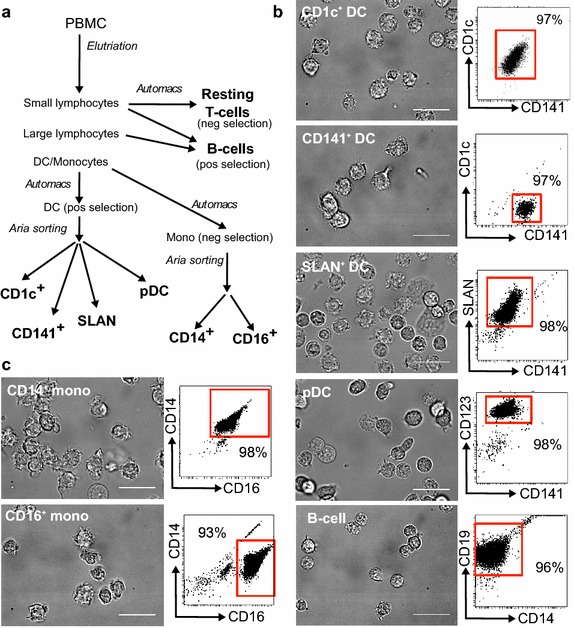
Isolation of antigen presenting cells. **a** Peripheral blood mononuclear cells (PBMC) were elutriated into three fractions: small lymphocytes, large lymphocytes and a monocyte/DC fraction. Resting CD4^+^ T-cells were isolated from the small lymphocyte fraction by negative selection using magnetic beads. Bulk B-cells were isolated from a mixture of the small and large lymphocyte fractions using positive magnetic bead selection for CD19. Bulk DC subpopulations were positively selected on the basis of expression of CD1c, CD141, SLAN and CD123 from the DC/monocyte fraction using magnetic bead selection. The positive “DC enriched” (DC) population was then sorted by flow cytometry into the four DC populations (purity >95 %). The negative “DC depleted” (mono) fraction was labeled with the monocyte markers CD14 and CD16, positively selected using magnetic beads and further sorted by flow cytometry into CD14^+^ and CD14^lo^CD16^hi^ subsets (purity >90 %). **b**, **c** Representative *dot plots* and brightfield images show the purity and morphology of the sorted APC subpopulations, respectively. The *scale bars* represent 20 μm, images were annotated using ImageJ software

Given that we were able to show induction of latency in non-proliferating CD4^+^ T-cells following co-culture with both bulk monocytes and mDC, we next compared the latency inducing potential of the different monocyte and mDC subpopulations. Monocytes were sorted into CD14^+^ and CD14^lo^CD16^+^ (CD16^+^) cells and mDC were sorted into CD1c^+^, CD141^+^ and SLAN^+^ DC, B-cells and pDC were also isolated by sorting (Fig. 2a). The final purity for all sorted APC subpopulations was >90 %, as determined post-sort by expression of specific known surface markers for the various subpopulations [26–30]. The APC subpopulations were examined using brightfield microscopy after culture (Fig. 2b, c). The mDC and monocyte subpopulations were characterized with the formation of both long and short dendritic processes (Fig. 2b, c) Comparatively, pDC and B-cells had few processes or ruffles (Fig. 2b, c; [28, 29, 31–33]).

**Fig. 3:**
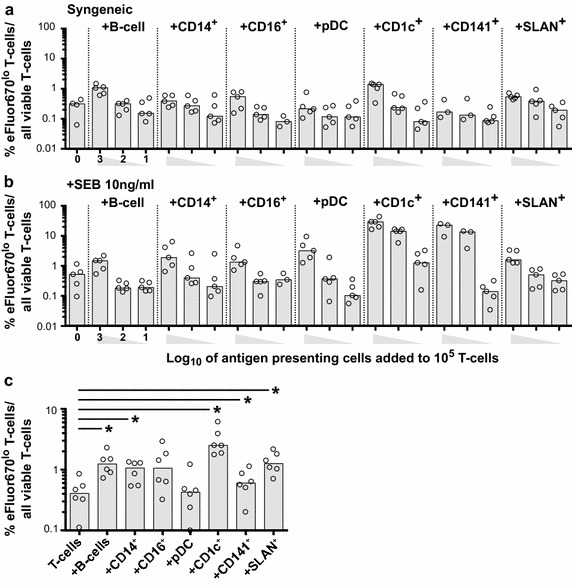
Resting CD4^+^ T-cell stimulation following co-culture with antigen presenting cells. Resting CD4^+^ T-cells were labeled with the proliferation dye eFluor670 and co-cultured with one of seven antigen presenting cell (APC) subpopulations, including B-cells; monocyte subpopulations-CD14^hi^ and CD14^lo^CD16^hi^; DC subpopulations- plasmacytoid (p)DC and myeloid (m)DC subpopulations—CD1c^+^, CD141^+^ and SLAN^+^, at a ratio of log 1 (10:1), 2 (100:1) or 3 (1000:1) T-cells : APC. T-cell stimulation was measured by quantification of the percentage of eFluor670^lo^ CD4^+^ T-cells from APC-T-cell co-cultures following 5 days of culture in the **a** absence (syngeneic) or **b** presence of staphylococcal enterotoxin B (SEB). **c** eFluor670 labeled, resting CD4^+^ T-cells were cultured alone, or with APC subpopulations at a ratio of 10:1 and infected with NL(AD8)Δnef-EGFP. At day 3 post-infection, CD4^+^ eFluor670^lo^ T-cells were measured. Columns represent the median, open circles represent individual donors, *p ≤ 0.05, as determined by Wilcoxon matched pairs signed rank test

APC function was tested in a syngeneic mixed leukocyte reaction (MLR) using the proliferation dye eFluor670 to measure proliferation of resting CD4^+^ T-cells. In the absence of mitogen stimulation, the relative potency of the various APC to induce T-cell proliferation at a ratio of 1 APC:10 CD4^+^ T-cells is shown (Fig. 3a). CD1c^+^ DC were the most potent at activating resting CD4^+^ T-cells, while pDC and CD141^+^ DC were least potent. The use of superantigen staphylococcal enterotoxin B (SEB) at low dose in the MLR had a modest effect on enhancing the capacity of APC to induce T-cell proliferation. T-cell proliferation following co-culture and SEB treatment was highest with CD1c^+^ DC and lowest with B-cells (Fig. 3b), confirming previous observations by others [26]. B-cells had a similar stimulatory capacity with and without superantigen (1.0 and 1.3 % proliferated CD4^+^ T-cells). Finally, there was a dose response of CD4^+^ T-cell proliferation with decreasing APC:T-cell ratio (1:10–1000). Together, these data confirm that all the APC subpopulations isolated remained functional in the co-cultures used for infection.

### T-cell stimulation by APC subpopulations in HIV-1 infected co-cultures

We then measured T-cell proliferation following co-culture with different APC subpopulations at 3 days following HIV-1 infection. The pattern of APC potency in induction of CD4^+^ T-cell proliferation in the presence of HIV-1 was similar to uninfected, sygeneic, co-cultures (Fig. 3c), where proliferation of CD4^+^ T-cells was highest with CD1c^+^ DC and lowest with pDC. These experiments demonstrate that HIV-1 infection did not independently alter APC or T-cell function with respect to T-cell proliferation.

### Several APC subpopulations enhanced productive infection of resting CD4^+^ T-cells

**Fig. 4:**
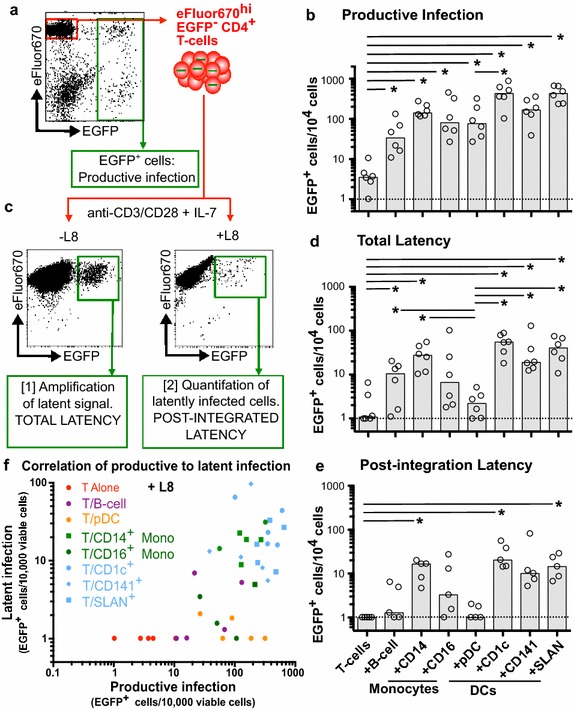
Productive and latent infection in resting T-cells co-cultured with antigen presenting cell subsets. **a** Representative dot plot of CD4^+^ T-cells co-cultured with antigen presenting cell (APC) subpopulations day 5 post infection with NL(AD8)Δnef-EGFP. Non-proliferating (eFluor670^hi^), non-productively infected (EGFP^−^) T-cells were sorted 5 days following infection. **b** EGFP expression in the total cell cultures at day 5 post-infection was used as a measure of productive infection. **c** Total and post-integrated latency was stimulated from eFluor670^hi^EGFP^−^ cells with anti-CD3/CD28 and IL-7 for 3 days and EGFP expression was quantified using flow cytometry. Representative *dot plots* show EGFP expression following stimulation of eFluor670^hi^EGFP^−^ sorted CD4^+^ T-cells in the absence (-L8 plot; total latency) and presence (+L8 plot; post integration latency) of the integrase inhibitor L8. **d** The frequency of total and **e** post-integration latent infection in resting CD4^+^ T-cells following co-culture with APC subpopulations. **f** Correlation of the frequency of productive infection and post-integrated latency (+L8) in each APC-T-cell co-culture. Each condition is identified by a *different color* and/or *symbol*. For all other panels, columns represent the median and open circles represent results from individual donors. Significant differences between conditions was measured by Wilcoxon matched pairs signed rank test where *p ≤ 0.05

We tested the ability of APC subpopulations to induce both productive and latent infection in resting CD4^+^ T-cells when cultured alone or co-cultured with one of the seven sorted APC subpopulations (Fig. 2a). Five days following infection, EGFP expression was quantified by flow cytometry as a measure of productive infection (Fig. 4a). We observed a significant increase in productive infection following HIV-1 infection in all APC co-cultured with T-cells compared to resting CD4^+^ T-cells cultured alone (p = 0.03 for all APC co-cultures; Fig. 4b).

### Different APC subpopulations can effectively induce latent infection in non-proliferating CD4^+^ T-cells

Five days following infection, non-proliferating (eFluor670^hi^EGFP^−^) CD4^+^ T-cells were sorted from the APC-T-cell co-cultures to quantify latent infection (Fig. 4a). The sorted CD4^+^ T-cells were directly stimulated with anti-CD3/CD28 and IL-7 (Fig. 4c) in the presence and absence of an integrase inhibitor, L8. EGFP was quantified by flow cytometry as a measure of inducible latent infection. Total latent infection (no L8) was significantly increased in non-proliferating CD4^+^ T-cells co-cultured with all mDC subpopulations, CD14^+^ monocytes and B-cells, when compared to CD4^+^ T-cells cultured alone (p = 0.03; Fig. 4d). In comparison, total latent infection following co-culture with CD14^lo^CD16^+^ monocytes, that were depleted of SLAN^+^ DC, was highly variable and not significantly different to T-cells cultured alone. As previously shown, latent infection was not found in T-cells co-cultured with pDC (p = 0.03 compared to mDC co-cultures; Fig. 4d).

We also quantified post-integration latent infection by stimulating T-cells with anti CD3/CD28 and IL-7 stimulation in the presence of L8. (Fig. 4c). The integrase inhibitor, L8, prevented any progression of pre-integration complexes to integration and inhibited secondary rounds of infection. Following infection of CD4^+^ T-cells co-cultured with each APC subpopulation, post-integration latency followed a similar pattern to that observed for total latency, but at a lower frequency (Fig. 4d, e). Post-integration latency was significantly increased in CD4^+^ T-cells following co-culture with mDC subpopulations CD1c^+^ and SLAN^+^, and CD14^+^ monocytes (p = 0.03, 0.02 and 0.01, respectively; Fig. 4e). Post-integration latency induced by CD141^+^ DC was elevated, similar to what was induced by other mDC subsets, but this did not reach statistical significance. In comparison, HIV-1 infection of T-cells co-cultured with SLAN DC depleted CD14^−^ CD16^+^ monocytes, B-cells and pDC was similar to infection of CD4^+^ T cells alone. Together these data show that only CD1c^+^mDC, SLAN^+^ DC and CD14^+^ monocytes were able to establish post-integration latent infection in non-proliferating CD4^+^ T-cells, while B-cells and CD141^+^ mDC were able to establish pre-integration latent infection. CD14^lo^CD16^hi^ SLAN^−^ monocytes, like pDC, were unable to establish either pre or post-integration latency.

Next, we looked for a correlation between productive infection and post-integration latency following infection of T-cells co-cultured with different APC (Fig. 4f). Overall, we found a weak correlation between productive and latent infection (Spearman’s r = 0.12; p = 0.02), which supports our previous findings [14]. However, the induction of productive infection does not inevitably lead to post-integration latency in resting CD4^+^ T-cells, as observed following co-culture with CD14^lo^CD16^hi^ monocytes, B-cells and pDC. We conclude that cells able to establish both productive and latent infection likely share common functional characteristics, which favour the establishment and maintenance of latent infection.

### Differential gene expression of cell-surface expressed molecules on APC

We next used RNA-seq to compare gene expression for genes involved in T-cell interactions with APC subpopulations that induce latency (CD1c^+^ DC, SLAN^+^ DC and CD14^+^ monocytes) compared to APC that could not (pDC). Due to difficulties isolating APC from T-cell co-cultures and HIV-1 infection, gene expression analysis was performed on freshly isolated APC subpopulations [34–36]. Component analysis showed clustering of the SLAN DC and CD14 monocytes and separate clusters of pDC and mDC (Additional file 1: Figure S1). Given that we have previously shown that cell contact is important in mDC-induced latency [14], we specifically selected genes encoding proteins that mediate mDC-T-cell interactions, including those in cell membrane compartments at the cell surface, and in intracellular vesicles such as endosomes and compartments giving rise to exosomes.

**Fig. 5:**
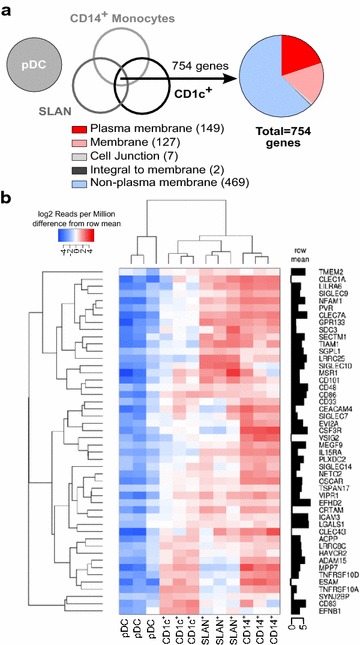
Comparison of gene expression between latency inducing and non-inducing antigen presenting cell subpopulations using RNA-seq. **a** Gene expression profiles common to the latency inducing APC subpopulations (CD1c^+^ mDC, SLAN^+^ mDC and CD14^+^ monocytes) compared with non-latency inducing APC (pDC) were selected (fold change ≥2, p < 0.01). Using GeneCodis, these 754 genes were categorised according to cellular compartment expression. **b** Encoded proteins expressed on APC surface and membrane compartments were further analysed for ability to signal to T-cells and involvement in HIV-1 infection. Representative heat map with >twofold differential gene expression of genes able to signal to T-cells, which are differentially expressed between latency inducing (CD1c^+^ mDC, SLAN^+^ mDC and CD14^+^ monocytes) and non-inducing APC subpopulations (pDC)

In APC subpopulations that induced post-integration HIV-1 latency compared to APC that didn’t induce latency, we found 754 differentially upregulated genes (fold change ≥2, p-value <0.01; Fig. 5a). Analysis for expression in cellular compartment (GeneCodis; http://genecodis.cnb.csic.es), identified 285 known genes expressed in: membrane, plasma membrane, integral to membrane, integral to plasma membrane and cell junction ([37–39]; Fig. 5a). Of these, 53 protein-encoding genes that could establish cell contact with CD4^+^ T-cells and potentially induce T-cell signaling were selected (Table 1, Additional file 2: Table S1). Functionally these genes included; cellular adhesion (32 genes), antigen presentation (1 gene), T-cell activation (9 genes), immune checkpoints (5 genes), regulation of apoptosis (5 gene), and an unknown protein (1 genes). We further analysed the role of each gene in HIV-1 infection of DC and CD4^+^ T-cells using a PubMed search for the interactions between DC and T-cells, and potential roles in the establishment of HIV-1 latency (Additional file 2: Table S1).

**Table 1:** Effects on HIV infection of genes differentially expressed by latency inducing and non-inducing antigen presenting cell subpopulations using RNA-seq

Gene name	Gene symbol	Function					
		Antigen presentation	Apoptosis regulation	Cell proximity presentation	Immune checkpoint blocker	T-cell activation	Unknown
Number of genes expressed in each category		1	5	32	5	9	1
*CD1d molecule*	*CD1d*	–^*,**^					
Lectin, galactoside-binding, soluble, 1	LGALS1		+				
Vasoactive intestinal peptide receptor 1	VIPR1		+^*^				
EF-hand domain family, member D2	EFHD2		–				
Tumor necrosis factor receptor superfamily, member 10a	TNFRSF10A		+				
Tumor necrosis factor receptor superfamily, member 10d, decoy with truncated death domain	TNFRSF10D		+				
Acid phosphatase, prostate	ACPP			+			
ADAM metallopeptidase domain 15	ADAM15			+			
Integrin, beta 2 (complement component 3 receptor 3 and 4 subunit)	CD18			+^*,**^			
Carcinoembryonic antigen-related cell adhesion molecule 4	CEACAM4			+			
C-type lectin domain family 4, member G	CLEC4G			–^*,**^			
*C*-*type lectin domain family 7, member A*	*CLEC7A*			+^*^			
Cytotoxic and regulatory T cell molecule	CRTAM			–^**^			
Colony stimulating factor 3 receptor (granulocyte)	CSF3R			+^*^			
Ephrin-B1	EFNB1			–			
*Endoglin*	*END*			+^*^			
Endothelial cell adhesion molecule	ESAM			+			
G protein-coupled receptor 133	GPR133			+			
*Intercellular adhesion molecule 3*	*ICAM3*			+^*^			
Leucine rich repeat containing 8 family, member C	LRRC8C			+			
Multiple EGF-like-domains 9	MEGF9			+			
Membrane protein, palmitoylated 7 (MAGUK p55 subfamily member 7)	MPP7			+			
Macrophage scavenger receptor 1	MSR1			+			
Osteoclast associated, immunoglobulin-like receptor	OSCAR			+			
Plexin domain containing 2	PLXDC2			+			
Syndecan 3	SDC3			+^*^			
CD33 molecule	CD33			–	–		
Sphingosine-1-phosphate lyase 1	SGPL1			+	+		
*Sialic acid binding Ig*-*like lectin 10*	*SIGLEC10*			–^*^	–		
Sialic acid binding Ig-like lectin 7	SIGLEC7			+^**^	+		
Sialic acid binding Ig-like lectin 9	SIGLEC9			–	–		
Synaptojanin 2 binding protein	SYNJ2BP			+	+		
T-cell lymphoma invasion and metastasis 1	TIAM1			+^*^	+		
Transmembrane protein 2	TMEM2			+	+		
Tetraspanin 17	TSPAN17			–	–		
C-type lectin domain family 1, member A	CLEC1A			+		+	
Sialic acid binding Ig-like lectin 14	SIGLEC14			+		+	
CD101 molecule	CD101				–		
CD52 molecule	CD52		–		–		
Hepatitis A virus cellular receptor 2	HAVR2/Tim-3				–^**^		
Leukocyte immunoglobulin-like receptor, subfamily A (with TM domain), member 6	LILRA6				–		
Poliovirus receptor	PVR				+^*^		
Neuropilin (NRP) and tolloid (TLL)-like 2	NETO2				+		
CD48 molecule	CD48					0^**^	
Interleukin 15 receptor, alpha	IL15RA					–^**^	
Leucine rich repeat containing 25	LRRC25					+	
NFAT activating protein with ITAM motif 1	NFAM1					+	
Secreted and transmembrane 1	SECTM1					+	
V-set and immunoglobulin domain containing 2	VSIG2					+	
CD300e molecule	CD300e					+	
CD83 molecule	CD83					0^**^	
CD86 molecule	CD86					0^**^	
Ecotropic viral integration site 2A	EVI2A						+

The functional category shown were determined by the description from the DAVID (http://david.abcc.ncifcrf.gov/) and GeneCards (http://genecards.org/) databases

+, increased latent infection; –, inhibition of virus expression, 0, undefined. Genes that were common to the RNA-seq and microarray generated gene lists are in italics. * represent a role in HIV infection of either DC (*) or T-cell (**). Data in Table 1 is presented with additional detail and references in Additional file 3: Supplementary references

We performed the same comparisons between selected APC subpopulations using our previously published microarray data [40] and found 27 genes that could potentially induce T-cell signalling (Additional file 4: Figure S2; Additional file 5: Table S2; [40]). Five of these genes were common between microarray and RNAseq analyses, seven genes shared protein family and thirteen genes fell outside of significance (cut off of p < 0.01), often with inconsistent replicates (n = 3). The common genes included *C*-*type lectin domain family 7 member A* (*CLEC*-*7A*), *endoglin, intracellular adhesion molecule 3* (*ICAM*-*3*), *sialic acid*-*binding immunoglobulin*-*type lectins* (*SIGLEC*)-*10* and *CD1d*. CD1d is involved in lipid antigen presentation to T-cells, while the other 4 surface expressed proteins are involved in cellular adhesion [41]. The common protein families included the SIGLEC family, CLEC family, leukocyte associated immunoglobulin like receptor (LILRA) family, G-Protein coupled receptor (GCPR) family and the tumor necrosis factor (TNF) receptor superfamily.

## Discussion

Latently infected cells are infrequent in HIV-1-infected patients on cART, and therefore robust in vitro models are needed to better understand the establishment and maintenance of latent infection. We have now shown that multiple APCs, in addition to mDC, are able to induce HIV-1 latency in non-proliferating CD4^+^ T cells [14]. Here, we have shown that the myeloid lineage cells capable of producing latent T-cell infection include subpopulations of blood derived mDC; CD1c^+^, SLAN^+^ DC and CD14^+^ monocytes, and confirmed that pDC are distinct in not generating latent infection. We have used RNA-seq analysis to define genes differentially expressed between APC subpopulations that could (CD1c^+^, SLAN^+^, CD14^+^ monocytes) and could not induce latent infection (pDC), and identified genes mediating cell adhesion, T-cell activation, immune checkpoints (IC) and regulation of apoptosis as important pathways differentially upregulated in the APC that are able to induce latent infection.

Our results show that multiple blood derived mDC subsets can induce latent infection in non-proliferating CD4^+^ T-cells, suggesting that this observation may extend to other DC and myeloid lineage cells, such as LC and dermal DC (DDC) though they may have different ontogeny [42–44]. It is likely that mDC and monocyte lineage cells in lymphoid tissue, skin, mucosal surfaces, gastrointestinal tract (GIT) and sites of inflammation could allow seeding of CD4^+^ T-cell viral reservoirs early following infection or during ART in tissue sites were cART penetration may not be optimal [45].

We observed a trend between the ability of the different APCs to induce latent infection and efficient T-cell proliferation. This suggests that there may be a common mechanism for the induction of T-cell proliferation and induction of latent infection, even in non-proliferating cells, perhaps through a bystander mechanism. Efficient T-cell proliferation is favoured by the formation of an immunological synapse [46, 47] with cellular proximity [48], major-histocompatibility complex (MHC)-T-cell receptor (TcR) interaction and co-stimulation [49–52]. ICAM-1 interaction with leukocyte function-associated antigen (LFA)-1 can facilitate induction of latent infection in the DC-T-cell model [14], while in other models of in vitro latency CD2 expression, a molecule that binds to LFA-3, was increased on latently infected cells [53]. A large portion (60 %) of genes identified in the gene expression analysis mediates cellular proximity and cell adhesion. Taken together these data suggest that cell adhesion/contact is important in the induction of latency. However, identification of a single specific adhesion molecule critical for the induction of latent infection is likely limited by functional redundancy in mediators of APC-T-cells interactions.

Compared to the other DCs, the pDC were least efficient for T-cell proliferation and induced significantly less productive and latent infection compared to mDC. The differences between mDC and pDC in induction of productive infection [54, 55] and suppression of virus production has been observed previously [14]. We have also previously shown that pDC were unable to induce HIV-1 latency, and that there was a more substantial suppressive effect on the establishment of latency compared to productive infection. We and others have shown multiple differences between mDC and pDC that may reduce the ability of pDC to establish close interactions with T-cells [26, 40, 56, 57], which in combination with increased type-I IFN secretion from pDC may inhibit the capacity of pDC to establish latency in T-cells.

The ability of B-cells to induce latent infection in non-proliferating CD4^+^ T-cells was also tested in this study as B-cells express MHC-II, circulate through LN, and have been reported to transfer HIV-1 infection to T-cells [58]. Induction of latency occurred only at low level and was only in the form of pre-integration latency suggesting that B-cells lack factors that facilitate efficient induction of post-integration latency.

Comparison of APC subpopulations that could and could not induce latent infection in non-proliferating CD4^+^ T-cells identified several functions that may be important in the establishment of latency, including cell adhesion, IC, T-cell co-stimulation, antigen presentation and regulation of apoptosis. The IC, programmed death receptor (PD)-1, is proposed to play a role in the establishment and/or maintenance of HIV-1 latency [59, 60]. Engagement of ICs, led to reduced T-cell activation by inhibition of signaling cascades, as well as physical inhibition of the formation of lipid rafts and cellular interactions [61]. In this study, we observed an up-regulation of the ICs; *CD101, T*-*cell immunoglobulin mucin*-*3* (*Tim*-*3, HAVR2*), *leukocyte immunoglobulin*-*like receptor member 6* (*LILR6*) and *CD52*, on latency inducing APC subpopulations when compared to pDC. IC expression may be important for the establishment of HIV-1 latency in this model, but further work is required to confirm this.

Additionally, we identified differential expression of the SIGLEC family of proteins between APC subpopulations that could and could not induce latent infection. We specifically found *SIGLEC 5, 7, 9, 10* and *14* to be upregulated on latency inducing APCs. From this family, SIGLEC 3, 5-11 have all been implicated in the inhibition of T-cell activation [62–64]. SIGLEC 5 has been shown to inhibit T-cell activation in chimpanzees, where blockade of SIGLEC 5 led to increased T-cell activation, and transfection of SIGLEC 5 into SIGLEC negative cells reduced T-cell activation [64–67]. SIGLEC 10 is hypothesized to have similar function in inhibition of T-cell activation [68, 69]. Together these data suggest that SIGLEC 5 or 10 binding to its ligand on the CD4^+^ T-cell may reduce T-cell activation, reduce productive infection and potentially promote latent infection. This is a novel association but further work will be required to explore any direct effects of SIGLEC proteins and the establishment of latency.

## Conclusion

This study has established that multiple myeloid lineage APC subpopulations can facilitate latent infection in resting CD4^+^ T-cells. Particularly important is the observation that CD14^+^ monocytes can induce latent infection in resting CD4^+^ T-cells. The use of CD14^+^ monocytes will greatly enhance the utility of this model. In addition, through a comparative analysis of APC populations, we have identified new pathways that may potentially be involved in the establishment and/or maintenance of HIV-1 latency. Inhibition of key pathways involved in mDC-T-cell interactions and HIV-1 latency may provide novel targets to eliminate HIV-1 latency.

## Methods

### Isolation and preparation of resting CD4^+^ T-cells and B-cells

PBMC were isolated by Ficoll-Paque density gradient centrifugation (GE Healthcare, Chalfont St. Giles, UK) from healthy buffy coats obtained from the Australian Red Cross. PBMC were further separated into three populations by counter-current elutriation using Beckman J-6M/E centrifuge equipped with a JE 5.0 rotor (Beckman Coulter, Pasedena, CA, USA; [70]). The three fractions were isolated at rates of 12 (small lymphocytes), 16 (large lymphocytes) and 20 (DC/Monocytes fractions) ml/min. Resting CD4^+^ T-cells, negative for the activation markers CD69 and HLA-DR, were sorted from the “small lymphocyte” fraction, as previously described [14], with a purity always >98 %. B-cells were isolated with a purity of ≥90 % from the “small and large lymphocyte” fractions using positive magnetic bead selection on an autoMACS (Miltenyi) using anti-CD19^+^ hybridoma (clone FMC63) and anti-IgG microbeads (Miltenyi, Bergisch Gladbach, Germany).

### Isolation of DC and monocytes

The remaining elutriated fraction, containing the larger cells (20 ml/min), was used to isolate DC and monocytes. The large cell fraction was first stained with antibodies specific for the DC subsets, which included CD1c-APC (Miltenyi), CD141-VioBlue (Miltenyi), CD123-PE (BD BioSciences, Franklin Lakes, NJ, USA) and SLAN-FITC (Miltenyi), and labeled with anti-IgG beads (Miltenyi). DC were then isolated using an AutoMACS (Miltenyi) into positive and negative fractions. The positive fraction (DC enriched) was further sorted into four DC subsets: CD1c^+^ mDC, SLAN^+^ DC, CD141^+^ mDC and CD123^+^ pDC, using a FACSAria (BD BioSciences). The negative fraction (DC depleted/mono) was stained with anti-CD14-FITC and anti-CD16-PE (BD Biosciences) antibodies, labeled with IgG beads (Miltenyi) and a positive selection performed using an AutoMACS (Miltenyi) to obtain a bulk monocyte population. These cells were further sorted to obtain the CD14^+^CD16^−^ (CD14^+^) and CD16^+^CD14^lo^ (CD16^+^) monocyte subsets using a FACSAria. Cell populations with a purity ≥90 % were used, as determined by flow cytometry (LSR II or FACSAria; BD Bioscience). In the event of low yields of some APC subpopulation, the experiment was continued without that population. In these experiments the missing data was omitted from the plots and therefore not every donor has data shown for all conditions tested.

### Imaging antigen presenting cell subpopulations

After isolation, each antigen presenting cell (APC) subpopulation was cultured in RF10 media (RPMI 1640; Life Technologies, Carlsbad, CA, USA), supplemented with 10 % fetal bovine serum (FBS; Interpath, Heidelberg, Australia), Penicillin–Streptomycin-Glutamine (PSG; Life Technologies) for 1–2 h at 37 °C in glass-bottom imaging plates (μ-slide, ibidi, Martinsried, Germany). Ten representative images were captured on a CCD camera through a 10 × 0.3 NA lens on a Olympus IX51 microscope and annotated with ImageJ software.

### Syngeneic mixed leukocyte reactions

Resting CD4^+^ T-cells were labeled with eFluor670 and co-cultured with decreasing concentrations of each APC subpopulation; log 1 (10:1), 2 (100:1) and 3 (1000:1), in the absence (syngeneic) or presence of superantigen SEB (10 ng/mL; Sigma). At day 5, cells were harvested and labeled with antibody against CD3 (V450, BD Bioscience). Cells were analysed by flow for T-cells that proliferated and therefore expressed low levels of eFluor670.

### Viral plasmids, virus preparation and infection

In all experiments, we used HIV-1 NL4.3 plasmid backbone with an AD8 envelope and EGFP inserted in the *nef* open reading frame at position 75 (NL(AD8)Δ*nef*EGFP) [14], kindly provided by Damian Purcell, University of Melbourne (Melbourne, Australia). Viral stocks were generated by FuGene (Promega, Madison, WI, USA) transfection of 293T cells as previously described [14]. Cells were infected at an MOI of 0.5, as determined by limiting dilution in PHA-stimulated PBMC using the Reed and Muench method [71].

### In vitro latency model

Resting CD4^+^ T-cells were labeled with the proliferation dye eFluor670 and cultured alone or with one of seven sorted syngeneic APC subpopulations at a ratio of 10:1 for 24 h in IL-2 (2U/mL, Roche Diagnostics, Basel, Switzerland) supplemented RF10 media. APC included monocyte subpopulations (CD14^+^CD16^−^ and CD14^lo^CD16^+^), DC subpopulations (pDC, CD1c^+^, CD141^+^ and SLAN^+^), and B-cells. Co-cultures were then infected with NL(AD8)Δ*nef*EGFP for 2 h, after which time excess virus was washed away and cells were cultured for an additional 5 days. In order to compare APC stimulatory capacity between APC-T-cell co-cultures, at day 3 post-infection, cells were stained with anti-CD3-V450 (BD Biosciences) to differentiate between T-cell and APC, and the proportion of proliferated (eFluor670^lo^) CD4^+^ T-cells were determined. Day 3 was used because this is when productive infection reached is maximum and remained high until day 5 (unpublished data). Additional APC-T-cell ratio’s were not used due to low APC yields. At day 5 post-infection, productive infection was determined by EGFP expression and non-proliferating, non-productively infected (eFluor670^hi^ EGFP^−^) CD4^+^ T-cells were sorted using a FACSAria.

### Reactivation of latency from resting T-cells

Latent infection in the sorted, non-proliferating CD4^+^ T-cells (eFluor670^hi^EGFP^−^) was determined by comparison of stimulated with un-stimulated T-cells sorted from APC-T-cell co-cultures (control). 1x10^5^ sorted CD4^+^ T-cells were stimulated with immobilized anti-CD3 (7 μg/ml; Beckman Coulter), in RF10 media supplemented with CD28 (5 μg/mL; BD Biosciences), IL-7 (50 ng/mL; Sigma, St Louis, MO, USA), IL-2 (5U/mL; Roche), with (post-integrated latency) or without (total latency: pre- and post-integrated latency) integrase inhibitor L8 (1 μM; Merck, White House Station, NJ, USA). The concentration of L8 was determined previously by titration of L8 in phytohaemagglutinin (PHA; 10 μg/mL) activated PBMC infected with R5-EGFP virus at an MOI of 0.5, same concentration usedin co-cultures, and showed productive infection was completely blocked at 1 μM. This concentration used for all subsequent experiments. Cells were harvested after 72 h of stimulation and EGFP expression was quantified on the FacsCalibur (BD BioSciences).

In some experiments PHA (10ug/mL) and IL-2 (10 U/mL) stimulated feeder PBMC were used to activate T-cells as a measure of inducing virus replication form latency, as described previously [14].

### Cell preparation for next generation sequencing and generation of gene lists

APC from 3 donors were sorted as described above to obtain mDC subpopulations CD1c^+^, SLAN^+^, CD14^+^ monocytes and pDC which were immediately stored in RLT buffer (Qiagen, Limburg, The Netherlands). Total RNA was isolated from low cell number samples (<500,000 cells) using Qiagen ALL prep micro kits (Qiagen), while RNA from samples with >500,000 cells were isolated using Qiagen RNA easy mini kits (Qiagen), according to the manufacturer’s instructions. Total RNA content varied from 270.0 to 1879.7 ng.

The Australian Gene Research Facility Ltd (AGRF, Melbourne, Australia) prepared cDNA libraries, which were multiplexed on the Illumina HiSeq 2000 (Illumina, San Diago, CA, USA). For gene expression analysis, single reads were selected with 20 million reads of 50 bp read size gathered. The RNA-seq reads were aligned to the human reference hg19 using the TopHat2 aligner [72, 73] and quantified using htseq-count [74]. Mapping rates for RNA seq are shown (Additional file 6: Table S3). Differential expression was calculated using Voom/Limma [75] and visualization performed using Degust [76] (http://victorian-bioinformatics-consortium.github.io/degust/) and Vennt [77] (http://drpowell.github.io/vennt/). Genes with fewer than 10 reads across every sample were removed from the analysis.

APC subsets were categorized as latency-inducing and latency-non-inducing subsets. Using a fold change of greater than 2 and false discovery rate (FDR) of 0.01, we identified 754 genes that were significantly upregulated in latency inducing populations (CD1c^+^ mDC, CD14^+^ monocytes, SLAN^+^ DC) compared to latency non-inducing populations (pDC; Fig. 4). As direct cell contact is required for the establishment of mDC induced latency, only protein encoding genes from APC implicated in cell contact were selected using the GeneCodis database (http://genecodis.cnb.csic.es). We identified 285 genes from the initial list that encode for proteins known to be expressed on the plasma-membrane, membrane, integral to the plasma-membrane/membrane and cell junctions [37–39]. Finally, we manually curated this list to identify 53 genes known to be involved in T-cell signaling (Table 1; Additional file 1: Table S1). RNA-seq data is available through Gene Omnibus (GEO), serial number GSE70106.

As a comparison, we performed a similar analysis using our previously published microarray data using the same APC subpopulations [40]. Microarray data was kindly provided by Andrew Harman, Westmead Millennium Institute for Medical Research, Sydney University, Sydney [40]. The RNA extraction, labeling, hybridization, data processing, and analysis procedures used by Harman et al. are described previously for the cDNA gene array [78] and Illumina arrays [79]. Hybridization and data processing was performed by AGRF using sentrix human 6 v2 expression chips (Illumina).

### Ethics approval

The use of blood samples from normal donors for this study was approved by the Alfred Hospital (HREC 156/11) and Monash University (CF11/1888) Human Research and Ethics Committees. Donors were recruited by the Red Cross Blood Transfusion Service as normal blood donors and all provided written informed consent for the use of their blood products for the research.

### Statistical analysis

Differences between experimental conditions were analyzed using Wilcoxon matched pairs signed rank test (n ≥ 5) or paired student T-test (n < 5) on GraphPad Prism (Version 6). P-values ≤0.05 were considered significant. Differentially expressed RNA-seq and microarray genes were found to be significant using ANOVA [40].

## Additional files

**Additional file 1: Figure S1.** Multidimensional scaling (MDS) of sequenced APC subpopulations. RNA sequences were measured according to two dimensions, 1 (x-axis) and 2 (y-axis). Each dot represents an antigen presenting cell (APC) subpopulation sequence, as labeled, n = 3. Clustering of dots is indicative of similar gene expression profiles.
**Additional file 2: Table S1.** Comparison of gene expression between latency inducing and non-inducing antigen presenting cell subpopulations using RNA-seq. Using the bioinformatics databases DAVID [80], GeneCards and GeneCodis, cell compartment gene function was determine of each gene. Genes expressed on antigen presenting cell (APC)-surface with the ability to signal to T-cells were shortlisted and their role in HIV-1 infection and DC-T-cell interaction was further determined using PubMed. Genes that were common to the RNA-seq and microarray generated gene lists are in *italics* and underlined in Table 1. Acronyms used: intracellular adhesion molecules (ICAM), C-type lectin (CLEC), immunoglobulin (Ig), T-cell immune-receptor with Ig and tyrosine-based inhibition motif (ITIM) domains (TIGIT), DNAX accessory molecule-1 (DNAM-1), cytotoxic and regulatory T-cell molecule (CRTAM), junction adhesion molecules (JAMs), blood brain barrier (BBB), leukocyte function antigen (LFA), dendritic cell-specific intercellular adhesion molecule-3-grabbing non-integrin (DC-SIGN), galectin-1 (Gal-1), cysteine-dependent aspartate-directed proteases (caspase), factor for adipocyte differentiation 158 (FAD158), extracellular matrix (ECM), lymph node (LN), nuclear factor-kappa-B (NFkB), vascular endothelial growth factor (VEGF), major histocompatibility complex (MHC), T-cell receptor (TcR), cytotoxic T-lymphocyte-associated protein 4 (CTLA-4), TYRO protein tyrosine kinase-binding protein (TYROBP), interleukin (IL-), nuclear factor of activated T-cells (NFAT), vasoactive intestinal polypeptide receptor 1 (VIPR1), T-cell immunoglobulin mucin-3 (Tim-3), monocyte derived dendritic cells (MDDC), natural killer cells (NK), human T-lymphotropic virus (HTLV-1), NFAT activating protein with immune-receptor tyrosine-based activation motif (NFAM1, CNAIP), cortical thymocyte-like protein (CTH, CTXC), B-cell receptor (BcR), HIV-associated neurocognitive disorder (HAND), antibody dependent cellular cytotoxicity (ADCC), Fc receptor (FcR), scavenger receptor class-A-1 (SRA-1), epidermal growth factor like domain, multiple/protein 5/9 (EGFL5).
**Additional file 3: Supplementary references to Table 1.** Included is the literature documenting the association of specific genes found in this study and changes in HIV infection and expression.
**Additional file 4: Figure S2.** Differential gene expression assessed by microarray analysis between latency inducing and non-inducing antigen presenting cells. **A**. Microarray gene expression profiles of antigen presenting cell (APC) subpopulations that could induce latency (CD1c^+^, SLAN^+^, CD14^+^ monocytes) in non-proliferating CD4^+^ T-cells were compared with APC subpopulations that could not induce latency (plasmacytoid (p)DC). Genes that were expressed in all 3 latency inducing APC subpopulations, CD1c^+^, SLAN^+^, CD14^+^ monocytes, were categorized as candidate 3, genes expressed in only 2 APC subpopulations were categorized as candidate 2 and genes expressed only in 1 APC subpopulation were categorized as candidate 1. **B.** Using the bioinformatics databases DAVID, GeneCards and GeneCodis, Candidate 2 and 3 gene lists were analyzed for cellular compartment and function. Genes expressed on the APC cell surface, with the ability to signal to T-cells were shortlisted. **C.** Heat map shows differentially expressed genes with ≥ twofold differences between latency inducing APC subpopulations (CD14^+^ monocytes, CD1c^+^ mDC and SLAN^+^ mDC) and non-latency inducing (pDC).
**Additional file 5: Table S2.** Comparison of gene expression between latency inducing and non-inducing antigen presenting cell subpopulations using microarray. Using the bioinformatics databases DAVID, GeneCards and GeneCodis, gene expression compartment and function was determined. Genes expressed on the antigen presenting cell (APC)-surface with the ability to signal to T-cells were shortlisted.
**Additional file 6: Table S3.** Sequence mapping rates in RNA-seq.

## Abbreviations

APC: antigen presenting cell
cART: combination antiretroviral therapy
CLEC-7A: C-type lectin domain family 7 member A
DC: dendritic cells
DDC: dermal dendritic cell
EGFP: emerald-green fluorescent protein
FBS: fetal bovine serum
FDR: false discovery rate
FC: fold change
GCPR: G-coupled protein receptor
GEO: gene Omnibus
GIT: gastrointestinal tract
HAVR2: Hepatitis A virus cellular receptor 2
HIV-1: human immunodeficiency virus
ICAM: intracellular adhesion molecule 3
ICB: immune checkpoint blocker
IL-7: interluekin-7
IL-2: interluekin-2
LC: langerhan cell
LFA: leukocyte function associated antigen
LILR: leukocyte-associated immunoglobulin like receptor
LN: lymph node
mDC: myeloid dendritic cell
MHC: major histocompatibility complex
MLR: mixed leukocyte reaction
Mono: monocyte
PBMC: peripheral blood mononuclear cells
PD-1: programmed death receptor-1
pDC: plasmacytoid dendritic cell
PHA: phytohemagglutinin
SEB: staphylococcal enterotoxin B
SIGLEC: sialic acid-binding immunoglobulin-type lectins
SLAN: 6-sulfo LacNAc
Tim-3: T-cell immunoglobulin mucin-3
TNF: tumor necrosis factor
