# Supplementary figures and images for: The role of antigen presenting cells in the induction of HIV-1 latency in resting CD4+ T-cells
Source: Retrovirology. 2015 Sep 11;12:76. doi: 10.1186/s12977-015-0204-2 (PMC4567795; doi:10.1186/s12977-015-0204-2)

**A**

MDS Dimension 2

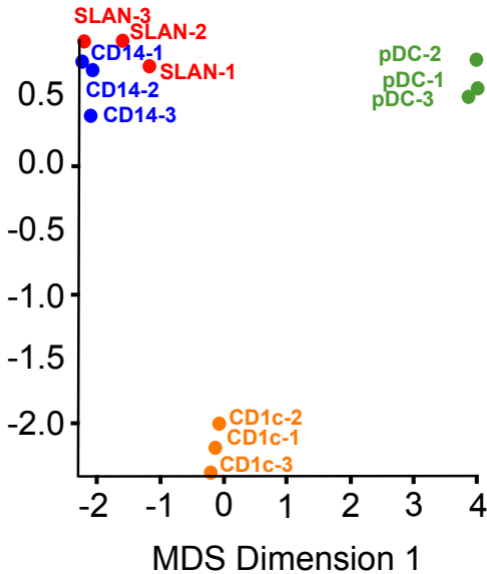

Supplement: Supplementary file 1 — Additional file 1: Figure S1. Multidimensional scaling (MDS) of sequenced APC subpopulations. RNA sequences were measured according to two dimensions, 1 (x-axis) and 2 (y-axis). Each dot represents an antigen presenting cell (APC) subpopulation sequence, as labeled, n = 3. Clustering of dots is indicative of similar gene expression profiles. [file 12977_2015_204_MOESM1_ESM.pdf]

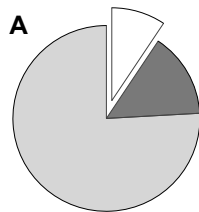

**Total genes = 1381**

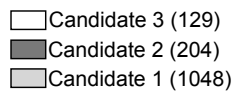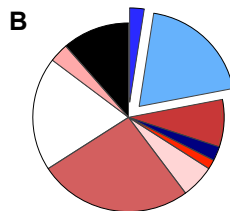

**Total genes = 123**

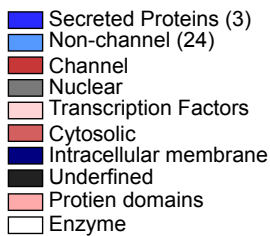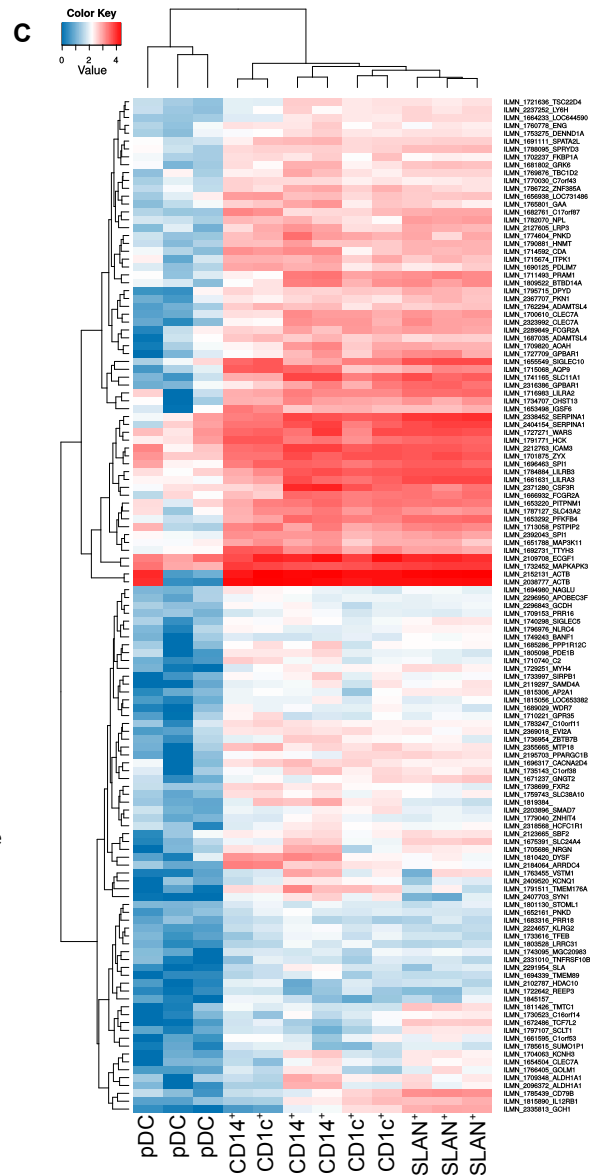

Supplement: Supplementary file 4 — Additional file 4: Figure S2. Differential gene expression assessed by microarray analysis between latency inducing and non-inducing antigen presenting cells. A. Microarray gene expression profiles of antigen presenting cell (APC) subpopulations that could induce latency (CD1c+, SLAN+, CD14+ monocytes) in non-proliferating CD4+ T-cells were compared with APC subpopulations that could not induce latency (plasmacytoid (p)DC). Genes that were expressed in all 3 latency inducing APC subpopulations, CD1c+, SLAN+, CD14+ monocytes, were categorized as candidate 3, genes expressed in only 2 APC subpopulations were categorized as candidate 2 and genes expressed only in 1 APC subpopulation were categorized as candidate 1. B. Using the bioinformatics databases DAVID, GeneCards and GeneCodis, Candidate 2 and 3 gene lists were analyzed for cellular compartment and function. Genes expressed on the APC cell surface, with the ability to signal to T-cells were shortlisted. C. Heat map shows differentially expressed genes with ≥ twofold differences between latency inducing APC subpopulations (CD14+ monocytes, CD1c+ mDC and SLAN+ mDC) and non-latency inducing (pDC). [file 12977_2015_204_MOESM4_ESM.pdf]
