# Supplementary material for: The role of antigen presenting cells in the induction of HIV-1 latency in resting CD4+ T-cells
Source: Retrovirology. 2015 Sep 11;12:76. doi: 10.1186/s12977-015-0204-2 (PMC4567795; doi:10.1186/s12977-015-0204-2)
Supplement: Supplementary file 3 — Additional file 3: Supplementary references to Table 1. Included is the literature documenting the association of specific genes found in this study and changes in HIV infection and expression. [file 12977_2015_204_MOESM3_ESM.doc]

***Table 1. Effects on HIV infection of genes differentially expressed by latency inducing and non-inducing antigen presenting cell subpopulations using RNA-seq.*** *The functional category shown were determined by the description from the DAVID (david.abcc.ncifcrf.gov/) and GeneCards (genecards.org/) databases.*

| **Gene name** | **Gene symbol** | **Function** | | | | | |
| --- | --- | --- | --- | --- | --- | --- | --- |
|  |  | **Antigen Presentation** | **Apoptosis regulation** | **Cell proximity presentation** | **Immune checkpoint blocker** | **T-cell activation** | **Unknown** |
| Number of genes expressed in each category |  | 1 | 5 | 32 | 5 | 9 | 1 |
| ***CD1d molecule*** | ***CD1d*** | *-^*,**^* |  |  |  |  |  |
| Lectin, galactoside-binding, soluble, 1 | LGALS1 |  | + |  |  |  |  |
| Vasoactive intestinal peptide receptor 1 | VIPR1 |  | +^*^ |  |  |  |  |
| EF-hand domain family, member D2 | EFHD2 |  | **-** |  |  |  |  |
| Tumor necrosis factor receptor superfamily, member 10a | TNFRSF10A |  | + |  |  |  |  |
| Tumor necrosis factor receptor superfamily, member 10d, decoy with truncated death domain | TNFRSF10D |  | + |  |  |  |  |
| Acid phosphatase, prostate | ACPP |  |  | + |  |  |  |
| ADAM metallopeptidase domain 15 | ADAM15 |  |  | + |  |  |  |
| Integrin, beta 2 (complement component 3 receptor 3 and 4 subunit) | CD18 |  |  | +^*,**^ |  |  |  |
| Carcinoembryonic antigen-related cell adhesion molecule 4 | CEACAM4 |  |  | + |  |  |  |
| C-type lectin domain family 4, member G | CLEC4G |  |  | -^*,**^ |  |  |  |
| ***C-type lectin domain family 7, member A*** | ***CLEC7A*** |  |  | *+^*^* |  |  |  |
| Cytotoxic and regulatory T cell molecule | CRTAM |  |  | -^**^ |  |  |  |
| Colony stimulating factor 3 receptor (granulocyte) | CSF3R |  |  | +^*^ |  |  |  |
| Ephrin-B1 | EFNB1 |  |  | - |  |  |  |
| ***Endoglin*** | ***END*** |  |  | *+^*^* |  |  |  |
| Endothelial cell adhesion molecule | ESAM |  |  | + |  |  |  |
| G protein-coupled receptor 133 | GPR133 |  |  | + |  |  |  |
| ***Intercellular adhesion molecule 3*** | ***ICAM3*** |  |  | *+^*^* |  |  |  |
| Leucine rich repeat containing 8 family, member C | LRRC8C |  |  | + |  |  |  |
| Multiple EGF-like-domains 9 | MEGF9 |  |  | + |  |  |  |
| Membrane protein, palmitoylated 7 (MAGUK p55 subfamily member 7) | MPP7 |  |  | + |  |  |  |
| Macrophage scavenger receptor 1 | MSR1 |  |  | + |  |  |  |
| Osteoclast associated, immunoglobulin-like receptor | OSCAR |  |  | + |  |  |  |
| Plexin domain containing 2 | PLXDC2 |  |  | + |  |  |  |
| Syndecan 3 | SDC3 |  |  | +^*^ |  |  |  |
| CD33 molecule | CD33 |  |  | - | - |  |  |
| Sphingosine-1-phosphate lyase 1 | SGPL1 |  |  | + | + |  |  |
| ***Sialic acid binding Ig-like lectin 10*** | ***SIGLEC10*** |  |  | *-^*^* | *-* |  |  |
| Sialic acid binding Ig-like lectin 7 | SIGLEC7 |  |  | +^**^ | + |  |  |
| Sialic acid binding Ig-like lectin 9 | SIGLEC9 |  |  | **-** | - |  |  |
| Synaptojanin 2 binding protein | SYNJ2BP |  |  | + | + |  |  |
| T-cell lymphoma invasion and metastasis 1 | TIAM1 |  |  | +^*^ | + |  |  |
| Transmembrane protein 2 | TMEM2 |  |  | + | + |  |  |
| Tetraspanin 17 | TSPAN17 |  |  | - | - |  |  |
| C-type lectin domain family 1, member A | CLEC1A |  |  | + |  | + |  |
| Sialic acid binding Ig-like lectin 14 | SIGLEC14 |  |  | + |  | + |  |
| CD101 molecule | CD101 |  |  |  | - |  |  |
| CD52 molecule | CD52 |  | - |  | - |  |  |
| Hepatitis A virus cellular receptor 2 | HAVR2  /Tim-3 |  |  |  | -^**^ |  |  |
| Leukocyte immunoglobulin-like receptor, subfamily A (with TM domain), member 6 | LILRA6 |  |  |  | - |  |  |
| Poliovirus receptor | PVR |  |  |  | +^*^ |  |  |
| Neuropilin (NRP) and tolloid (TLL)-like 2 | NETO2 |  |  |  | + |  |  |
| CD48 molecule | CD48 |  |  |  |  | 0^**^ |  |
| Interleukin 15 receptor, alpha | IL15RA |  |  |  |  | -^**^ |  |
| Leucine rich repeat containing 25 | LRRC25 |  |  |  |  | + |  |
| NFAT activating protein with ITAM motif 1 | NFAM1 |  |  |  |  | + |  |
| Secreted and transmembrane 1 | SECTM1 |  |  |  |  | + |  |
| V-set and immunoglobulin domain containing 2 | VSIG2 |  |  |  |  | + |  |
| CD300e molecule | CD300e |  |  |  |  | + |  |
| CD83 molecule | CD83 |  |  |  |  | 0^**^ |  |
| CD86 molecule | CD86 |  |  |  |  | 0^**^ |  |
| Ecotropic viral integration site 2A | EVI2A |  |  |  |  |  | + |

+ = increased latent infection, - =inhibition of virus expression, 0 = undefined. Genes that were common to the RNA-seq and microarray generated gene lists are in italics and **bold** * represent a role in HIV infection of either DC (*) or T-cell (**).(Münch et al. 2007; Colonna et al. 2000; Huysamen et al. 2009; de Witte et al. 2007; Arase et al. 2005; Li et al. 2004; Jaeger et al. 2010; Dunn et al. 2004; Govender et al. 2013; Ivanov & Romanovsky 2006; Deli 2009; Barat et al. 2004; Hahn et al. 2004; Mercier et al. 2008; Bhoj et al. 2009; Tan et al. 2013; Bobardt et al. 2007; Bobardt et al. 2003; Sensken et al. 2011; Spiegel & Milstien 2011; Soto et al. 2013; Angata et al. 2006; Adam et al. 2013; Chaudhry et al. 2007; Kelly et al. 2013; Chen et al. 2006; Moll et al. 2009; Ipp et al. 2014; Temerozo et al. 2013; Bokaei et al. 2007; Shankar et al. 2011; Jones et al. 2008; Anderson & Allen 2009; Chan et al. 2014; Li et al. 2014; Ghez et al. 2006; Matusali et al. 2012; Vassena et al. 2013; Lambert et al. 2009; Assarsson et al. 2005; Ward et al. 2007; Reinwald et al. 2008; Wilflingseder et al. 2004; Wang & Lewis 2001; Sabado et al. 2010; Sato et al. 2007; Mortier et al. 2009; Rissoan et al. 2002)

Supplementary references

Adam, M.G. et al., 2013. Synaptojanin-2 binding protein stabilizes the Notch ligands DLL1 and DLL4 and inhibits sprouting angiogenesis. *Circulation Research*, 113(11), pp.1206–1218.

Anderson, K.J. & Allen, R.L., 2009. Regulation of T-cell immunity by leucocyte immunoglobulin-like receptors: innate immune receptors for self on antigen-presenting cells. *Immunology*, 127(1), pp.8–17.

Angata, T. et al., 2006. Discovery of Siglec-14, a novel sialic acid receptor undergoing concerted evolution with Siglec-5 in primates. *FASEB journal: official publication of the Federation of American Societies for Experimental Biology*, 20(12), pp.1964–1973.

Arase, N. et al., 2005. Heterotypic interaction of CRTAM with Necl2 induces cell adhesion on activated NK cells and CD8+ T cells. *International Immunology*, 17(9), pp.1227–1237.

Assarsson, E. et al., 2005. 2B4/CD48-Mediated Regulation of Lymphocyte Activation and Function. *The Journal of Immunology*, 175(4), pp.2045–2049.

Barat, C., Gervais, P. & Tremblay, M.J., 2004. Engagement of ICAM-3 Provides a Costimulatory Signal for Human Immunodeficiency Virus Type 1 Replication in both Activated and Quiescent CD4+ T Lymphocytes: Implications for Virus Pathogenesis. *Journal of Virology*, 78(12), pp.6692–6697.

Bhoj, E.J. et al., 2009. MODY-like diabetes associated with an apparently balanced translocation: possible involvement of MPP7 gene and cell polarity in the pathogenesis of diabetes. *Molecular Cytogenetics*, 2, p.5.

Bobardt, M.D. et al., 2007. Cell-free human immunodeficiency virus type 1 transcytosis through primary genital epithelial cells. *Journal of Virology*, 81(1), pp.395–405.

Bobardt, M.D. et al., 2003. Syndecan Captures, Protects, and Transmits HIV to T Lymphocytes. *Immunity*, 18(1), pp.27–39.

Bokaei, P.B. et al., 2007. HIV-1 integration is inhibited by stimulation of the VPAC2 neuroendocrine receptor. *Virology*, 362(1), pp.38–49.

Chan, C.J. et al., 2014. The receptors CD96 and CD226 oppose each other in the regulation of natural killer cell functions. *Nature Immunology*, 15(5), pp.431–438.

Chaudhry, A. et al., 2007. A two-pronged mechanism for HIV-1 Nef-mediated endocytosis of immune costimulatory molecules CD80 and CD86. *Cell Host & Microbe*, 1(1), pp.37–49.

Chen, N. et al., 2006. HIV-1 down-regulates the expression of CD1d via Nef. *European Journal of Immunology*, 36(2), pp.278–286.

Colonna, M., Samaridis, J. & Angman, L., 2000. Molecular characterization of two novel C-type lectin-like receptors, one of which is selectively expressed in human dendritic cells. *European Journal of Immunology*, 30(2), pp.697–704.

Deli, M.A., 2009. Potential use of tight junction modulators to reversibly open membranous barriers and improve drug delivery. *Biochimica et Biophysica Acta (BBA) - Biomembranes*, 1788(4), pp.892–910.

Dunn, S.J. et al., 2004. Identification of cell surface targets for HIV-1 therapeutics using genetic screens. *Virology*, 321(2), pp.260–273.

Ghez, D. et al., 2006. Neuropilin-1 Is Involved in Human T-Cell Lymphotropic Virus Type 1 Entry. *Journal of Virology*, 80(14), pp.6844–6854.

Govender, N., Naicker, T. & Moodley, J., 2013. Maternal imbalance between pro-angiogenic and anti-angiogenic factors in HIV-infected women with pre-eclampsia. *Cardiovascular Journal of Africa*, 24(5), pp.174–179.

Hahn, H.P. et al., 2004. Galectin-1 induces nuclear translocation of endonuclease G in caspase- and cytochrome c-independent T cell death. *Cell Death and Differentiation*, 11(12), pp.1277–1286.

Huysamen, C., Brown, G.D. & Sullivan, D., 2009. The fungal pattern recognition receptor, Dectin-1, and the associated cluster of C-type lectin-like receptors. *Fems Microbiology Letters*, 290(2), pp.121–128.

Ipp, H. et al., 2014. CD4+ T cells in HIV infection show increased levels of expression of a receptor for vasoactive intestinal peptide, VPAC2. *Immunologic Research*, 60(1), pp.11–15.

Ivanov, A.I. & Romanovsky, A.A., 2006. Putative dual role of ephrin-Eph receptor interactions in inflammation. *IUBMB life*, 58(7), pp.389–394.

Jaeger, S. et al., 2010. Inference of Surface Membrane Factors of HIV-1 Infection through Functional Interaction Networks. *PLoS ONE*, 5(10), p.e13139.

Jones, R.B. et al., 2008. Tim-3 expression defines a novel population of dysfunctional T cells with highly elevated frequencies in progressive HIV-1 infection. *The Journal of Experimental Medicine*, 205(12), pp.2763–2779.

Kelly, H. et al., 2013. Effects of HIV-1-induced CD1c and CD1d modulation and endogenous lipid presentation on CD1c-restricted T-cell activation. *BMC immunology*, 14(4), pp.1–10.

Lambert, S. et al., 2009. HTLV-1 uses HSPG and neuropilin-1 for entry by molecular mimicry of VEGF165. *Blood*, 113(21), pp.5176–5185.

Li, M. et al., 2014. T-cell Immunoglobulin and ITIM Domain (TIGIT) Receptor/Poliovirus Receptor (PVR) Ligand Engagement Suppresses Interferon-γ Production of Natural Killer Cells via β-arrestin 2-mediated Negative Signaling. *Journal of Biological Chemistry*, p.jbc.M114.572420.

Li, Q. et al., 2004. Functional Genomic Analysis of the Response of HIV-1-Infected Lymphatic Tissue to Antiretroviral Therapy. *Journal of Infectious Diseases*, 189(4), pp.572–582.

Matusali, G. et al., 2012. The human immunodeficiency virus type 1 Nef and Vpu proteins downregulate the natural killer cell-activating ligand PVR. *Journal of Virology*, 86(8), pp.4496–4504.

Mercier, S. et al., 2008. Galectin-1 promotes HIV-1 infectivity in macrophages through stabilization of viral adsorption. *Virology*, 371(1), pp.121–129.

Moll, M. et al., 2009. Severe functional impairment and elevated PD-1 expression in CD1d-restricted NKT cells retained during chronic HIV-1 infection. *European Journal of Immunology*, 39(3), pp.902–911.

Mortier, E. et al., 2009. Macrophage- and dendritic-cell-derived interleukin-15 receptor alpha supports homeostasis of distinct CD8+ T cell subsets. *Immunity*, 31(5), pp.811–822.

Münch, J. et al., 2007. Semen-derived amyloid fibrils drastically enhance HIV infection. *Cell*, 131(6), pp.1059–1071.

Reinwald, S. et al., 2008. CD83 Expression in CD4+ T Cells Modulates Inflammation and Autoimmunity. *The Journal of Immunology*, 180(9), pp.5890–5897.

Rissoan, M.-C. et al., 2002. Subtractive hybridization reveals the expression of immunoglobulinlike transcript 7, Eph-B1, granzyme B, and 3 novel transcripts in human plasmacytoid dendritic cells. *Blood*, 100(9), pp.3295–3303.

Sabado, R.L. et al., 2010. Evidence of dysregulation of dendritic cells in primary HIV infection. *Blood*, 116(19), pp.3839–3852.

Sato, N. et al., 2007. The IL-15/IL-15R? on cell surfaces enables sustained IL-15 activity and contributes to the long survival of CD8 memory T cells. *Proceedings of the National Academy of Sciences of the United States of America*, 104(2), pp.588–593.

Sensken, S.-C. et al., 2011. Local Inactivation of Sphingosine 1-Phosphate in Lymph Nodes Induces Lymphopenia. *The Journal of Immunology*, 186(6), pp.3432–3440.

Shankar, E.M. et al., 2011. Expression of a broad array of negative costimulatory molecules and Blimp-1 in T cells following priming by HIV-1 pulsed dendritic cells. *Molecular Medicine (Cambridge, Mass.)*, 17(3-4), pp.229–240.

Soto, P.C. et al., 2013. Cell-intrinsic mechanism involving Siglec-5 associated with divergent outcomes of HIV-1 infection in human and chimpanzee CD4 T cells. *Journal of Molecular Medicine (Berlin, Germany)*, 91(2), pp.261–270.

Spiegel, S. & Milstien, S., 2011. The outs and the ins of sphingosine-1-phosphate in immunity. *Nature Reviews Immunology*, 11(6), pp.403–415.

Tan, R. et al., 2013. Nef interaction with actin compromises human podocyte actin cytoskeletal integrity. *Experimental and Molecular Pathology*, 94(1), pp.51–57.

Temerozo, J.R. et al., 2013. Macrophage Resistance to HIV-1 Infection Is Enhanced by the Neuropeptides VIP and PACAP. *PloS One*, 8(6), p.e67701.

Vassena, L. et al., 2013. The human immunodeficiency virus type 1 Vpr protein upregulates PVR via activation of the ATR-mediated DNA damage response pathway. *The Journal of General Virology*, 94(Pt 12), pp.2664–2669.

Wang, X. & Lewis, D.E., 2001. CD86 expression correlates with amounts of HIV produced by macrophages in vitro. *Journal of Leukocyte Biology*, 69(3), pp.405–413.

Ward, J. et al., 2007. HIV modulates the expression of ligands important in triggering natural killer cell cytotoxic responses on infected primary T-cell blasts. *Blood*, 110(4), pp.1207–1214.

Wilflingseder, D. et al., 2004. HIV-1-induced migration of monocyte-derived dendritic cells is associated with differential activation of MAPK pathways. *Journal of Immunology (Baltimore, Md.: 1950)*, 173(12), pp.7497–7505.

De Witte, L. et al., 2007. Syndecan-3 is a dendritic cell-specific attachment receptor for HIV-1. *Proceedings of the National Academy of Sciences of the United States of America*, 104(49), pp.19464–19469.
